# A Method for the Isolation of Exosomes from Human and Bovine Milk

**DOI:** 10.1155/2019/5764740

**Published:** 2019-12-03

**Authors:** Kanchan Vaswani, Murray D. Mitchell, Olivia J. Holland, Yong Qin Koh, Rebecca J. Hill, Tracy Harb, Peter S. W. Davies, Hassendrini Peiris

**Affiliations:** ^1^Institute of Health and Biomedical Innovation-Centre for Children's Health Research, Faculty of Health, Queensland University of Technology, Brisbane, Queensland 4029, Australia; ^2^University of Queensland Centre for Clinical Research, Faculty of Medicine, The University of Queensland, Brisbane, Queensland 4029, Australia; ^3^The University of Queensland, Brisbane, Queensland 4029, Australia

## Abstract

**Scope:**

Milk provides a natural means of nutrient supply to infants. Exosomes are an important component of milk that are not only being studied for their promise in translational medicine but also in infant nutrition. They also play important roles in intercellular communication and immune function in mammary glands and are able to transfer their materials to the recipient. Therefore, the isolation of high-quality exosomes is an important aspect of exosome research.

**Methods and Results:**

This study is a technical study, which provides a detailed methodology for the isolation and enrichment of exosomes from milk. In this study, we evaluate the suitability of using the exosome enrichment method that we have recently published for bovine milk, on human milk. We initially isolated extracellular vesicles from human and bovine milk on a fresh set of samples, using ultracentrifugation, and then exosomes were subsequently enriched via size exclusion chromatography (SEC). Following isolation and enrichment, exosomes from both species were characterized by particle concentration (nanoparticle tracking analysis, NTA), morphology (transmission electron microscopy, TEM), and the presence of exosomal markers (immunoblotting and mass spectrometry using information dependant acquisition (IDA)). The key exosomal characteristics of spherical/donut-shaped morphology, the presence of exosomal markers, e.g., FLOT-1 and the tetraspanins, CD9 and CD81), and particle concentration were confirmed in both human and bovine milk exosomes.

**Conclusion:**

We conclude that our robust exosome enrichment method, previously published for bovine milk, is suitable for use on human milk.

## 1. Introduction

Exosomes are a subtype of extracellular vesicles (EVs) that have a size range between 30 and 120 nm. These nanovesicles are found in many different biological fluids, including urine, plasma, saliva, and milk [1]. To date, there is no universally accepted methodology for the isolation of exosomes, and a number of methodologies have been published for each fluid type. Each isolation method has its limitations; for example, commercial exosome precipitation kits and ultracentrifugation and ultrafiltration techniques coprecipitate other nonexosomal contaminants such as proteins and macromolecules together with the exosomes they isolate [2].

The objective of this study was to evaluate the suitability of a method previously developed for the isolation of bovine milk exosomes for its application in the isolation of human milk exosomes. Our method uses the combination of differential ultracentrifugation and exosome enrichment by size exclusion chromatography (SEC) [1]. Ultracentrifugation at high speed is necessary to pellet the EVs while SEC is important as it subsequently separates particles by size [3]. The exosomes from both human and bovine milk were characterized by particle number (by nanoparticle tracking analysis, NTA), morphology (by transmission electron microscopy, TEM), and the presence of an exosomal protein marker FLOT-1 (by immunoblotting) and the two tetraspanins, CD9 and CD81 (by information dependant acquisition, IDA mass spectrometry).

## 2. Materials and Methods

### 2.1. Milk Collection

Human milk (9 ml) was collected from four healthy donor women in compliance with the University of Queensland Human Research Ethics Committee and the regulations governing experimentation on humans. Human milk (9 ml × 3) was used for subsequent experiments. Unpasteurised bovine milk (10 ml × 3) was collected from a healthy Holstein Friesian dairy herd located at Gatton, University of Queensland. Milk was aliquoted and stored at −80°C for later use.

### 2.2. Extracellular Vesicle Isolation and Exosome Enrichment

**Figure 1:**
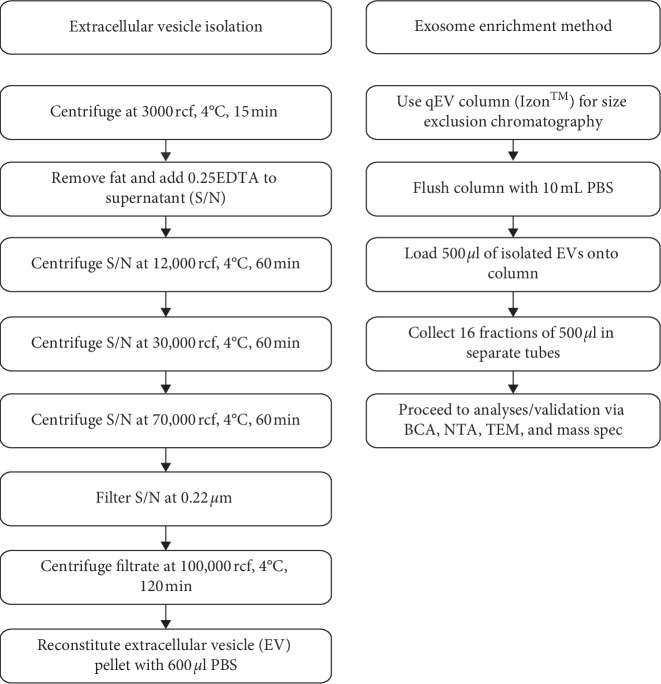
Flowchart showing methods for the isolation of extracellular vesicles (EVs) from milk and subsequent exosome enrichment. EVs were isolated from human and bovine milk by differential ultracentrifugation. Unpasteurised milk was centrifuged at 3000 rcf followed by 0.25M EDTA (1 : 1; v/v) treatment to remove excess casein and supernatants (S/N) subsequently centrifuged at 12,000, 30,000, 70,000, and 100,000 rcf, respectively. The pellet obtained after the sequential centrifugation process contains EVs. After reconstitution in PBS, the EV suspension was used for exosome enrichment. 600 *μ*l of EV suspension was introduced on top of a SEC column (qEV column) and processed via SEC to obtain 16 fractions as described in the flowchart.

EVs were isolated from milk by ultracentrifugation as previously published [1]. Briefly, bovine and human milk were centrifuged at 3000 and 12,000 rcf to remove fat globules, cellular debris, somatic cells, and casein. This was followed by centrifugation steps at high speed (Figure 1). The supernatants collected after removal of fat and casein were then filtered at 0.2 *µ*m (PES, Corning 431229, Sigma Aldrich, Castle Hill, NSW, Australia) and subjected to the final 100,000 rcf centrifugation step. The pellets containing the EVs were resuspended in 600 *μ*l PBS (Gibco, Life Technologies Australia Pty Ltd). 500 *μ*l of EVs was introduced on a washed qEV size exclusion chromatography column (qEV original SP1-AUD, Izon Science Ltd, New Zealand), and 500 *μ*l fractions were collected in 16 separate tubes as per manufacturer's instructions (Figure 1). Note that prior to starting SEC, a single representative column was used to test the chromatography principle using 100 nm latex beads (Malvern Panalytical Ltd, Malvern, UK) as a QC for separation of exosomes into fractions 7, 8, 9, and 10.

### 2.3. Nanoparticle Tracking Analysis

The number of exosomes isolated from both human and bovine milk was determined by nanoparticle tracking analysis (NTA) using a Malvern NanoSight NS500, NTA 3.0 instrument as per manufacturer's instructions. Pooled exosomal samples (i.e., fractions 7–10) were analysed (in triplicate) to determine particle concentration (particles/ml) in PBS. Then, the total yield was obtained by calculating the concentration in particles/ml multiplied with the total volume (2 ml) of exosomal fractions (i.e., 500 *µ*l fraction 7, 500 *µ*l fraction 8, 500 *µ*l fraction 9, and 500 *µ*l fraction 10). A Mann–Whitney *U* (unpaired) test was carried out between the two groups (*n* = 3 per group), for yield. The yield was then extrapolated back to the starting volumes of milk used (10 ml, bovine milk and 9 ml, human milk).

The NTA of the QC sample revealed particles in fractions 7–10.

### 2.4. Transmission Electron Microscopy

Human and bovine exosomal fractions (fractions 7–10) were pooled, and 5 *μ*l from each sample was analysed by transmission electron microscopy (TEM) by negative stain. Samples were placed on formvar-coated copper grids and viewed on a JEOL 1010 microscope [1].

### 2.5. Immunoblotting

Exosomal markers in both pooled samples and the individual fractions were determined by immunoblotting for FLOT-1 (details described subsequently). Exosomal protein concentration was quantified by using a Bicinchoninic Acid reagent kit (Sigma Aldrich, Castle Hill, NSW, Australia). 10 *μ*g of exosome protein (singular fractions and pooled fractions 7–10) was incubated for 10 min at 70°C in reducing agent (NuPAGE Sample Reducing Agent, Life Technologies Australia Pty Ltd, Mulgrave, VIC, Australia) and loading buffer (NuPAGE LDS buffer, Life Technologies Australia Pty Ltd). Reduced proteins were electrophoresed and transferred onto a polyvinyl difluoride (PVDF; Bio-Rad Laboratories Pty Ltd, Australia) membrane as previously described [1]. Membranes were incubated for 1 hour in 2% BSA and probed overnight with primary goat polyclonal antibody anti-Flotillin 1 (FLOT-1) (ab13493 Abcam, Cambridge, UK) at 4°C, followed by secondary donkey anti-goat IgG-HRP (1 : 1000; sc-2020, Santa Cruz Biotechnology, CA, USA). SuperSignal West Dura-Extended Duration Substrate (Thermo Fisher Scientific, Australia Pty Ltd) was used for development, and the signal visualized on X-ray film (Agfa, Mortsel, Belgium) was developed using a Konica Minolta SRX-101A processor (Konica Minolta Medical and Graphic Inc, Japan) (method derived from [1, 4]).

### 2.6. Mass Spectrometry Proteomic Profiling

Exosomal fractions (pooled fractions 7–10) of bovine and human samples were sonicated for 5 mins. 25 *µ*g of exosomal protein was used for mass spectrometry. Sample preparations were performed as previously published [1]. Eluted peptides were dried at RT in a vacuum evaporator for 45 min. Samples were then reconstituted in 30 *μ*l formic acid. The digested protein samples were analysed using the TripleTOF® 5600 mass spectrometer (ABSciex, Redwood City, CA) and Eksigent HPLC system to obtain initial high mass accuracy survey MS/MS data, identifying the peptides present. An in-depth proteomic analysis was performed using the IDA experiments on the TripleTOF® 5600 System interfaced with a nanospray source. Results were analysed using ProteinPilot™ (ABSciex, Redwood City, CA) (method derived from [1, 4]).

## 3. Results

### 3.1. Nanoparticle Tracking Analysis

**Figure 2:**
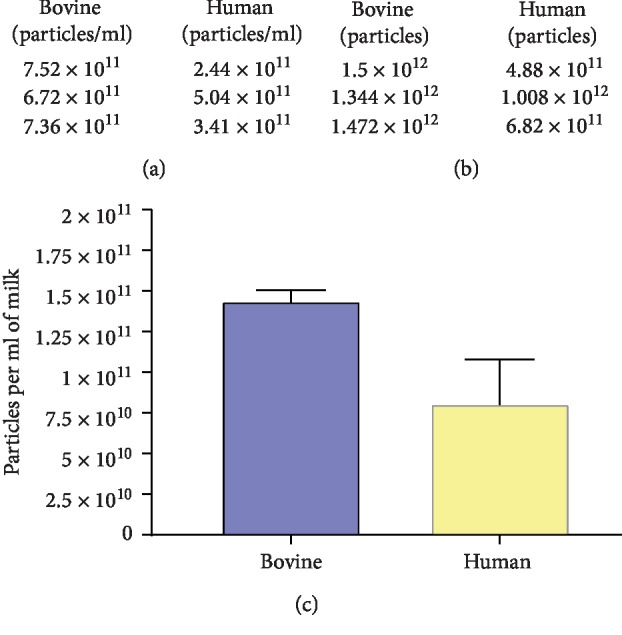
Nanoparticle tracking analysis (NTA) to determine the particle concentration (particles/ml), yield (particles), and particle concentration per volume of milk of the exosomes obtained after enrichment, for pooled fractions 7–10 (*n* = 3 experimental replicates). (a) The table indicates concentration (particles/ml) in PBS for both human and bovine milk exosomes (*n* = 3 per group). (b) The table depicts the total yield (particles) obtained from both bovine and human milk in a 2 ml exosome solution in PBS (*n* = 3 per group). (c) The figure displays concentration of exosome particles extrapolated to the initial volume of bovine and human milk (10 ml and 9 ml, respectively). The concentration is expressed as particles per ml of milk. The Mann–Whitney *U* (unpaired) test revealed no significant differences between the two groups (error bars ±SEM).

NanoSight™ measurements of pooled exosome samples indicate that human milk exosomes can be successfully quantified using the same technique as we developed for bovine milk exosomes. The average concentration of exosomes obtained was 7.2 × 10^11^ particles/ml (bovine) and 3.63 × 10^11^ particles/ml (human) (Figure 2(a)). The average yields of exosomes obtained was 1.44 × 10^12^ particles (bovine) and 7.26 × 10^11^ particles (human) (Figure 2(b)). Average particle concentration extrapolating to the starting volume of milk used was 1.4 × 10^11^ particles/ml of milk (bovine) and 8.0 × 10^10^ particles/ml of milk (human) (Figure 2(c)). The Mann–Whitney *U* (unpaired) test revealed no significant differences between the groups.

The NTA results of the QC sample successfully revealed particles in fractions 7–10 (more concentrated in fractions 8 and 9), but not in the later fractions as suggested by the manufacturer (data not shown).

### 3.2. Immunoblotting

**Figure 3:**
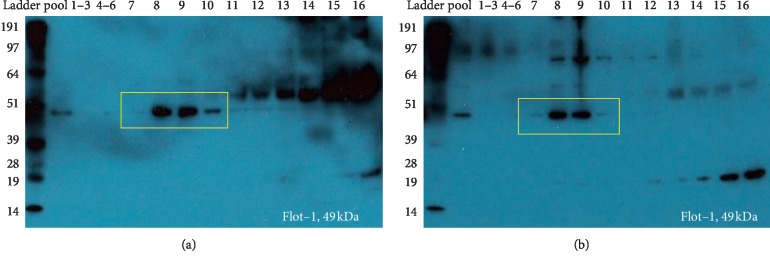
Immunoblotting for the presence of exosomal marker FLOT-1 (49 kDa). (a, b) The presence of FLOT-1 in exosomes in the pooled samples (fraction 7–10) for both human and milk exosome samples. The blots also indicate presence of FLOT-1 individual exosome fractions 7, 8, 9, and 10 for both species (highlighted in yellow box). The SEC void volumes (i.e., fractions 1–6) were pooled into two groups, F1–3 and F4–6, for ease of running SDS-PAGE on a single gel. (a) Bovine. (b) Human.

Immunoblotting of the pooled human exosome sample (fractions 7–10) and individual fractions 7–10 revealed presence of exosomal marker FLOT-1(49 kDa) similar to that of bovine exosomes (Figures 3(a) and 3(b)).

### 3.3. Transmission Electron Microscopy (TEM)

**Figure 4:**
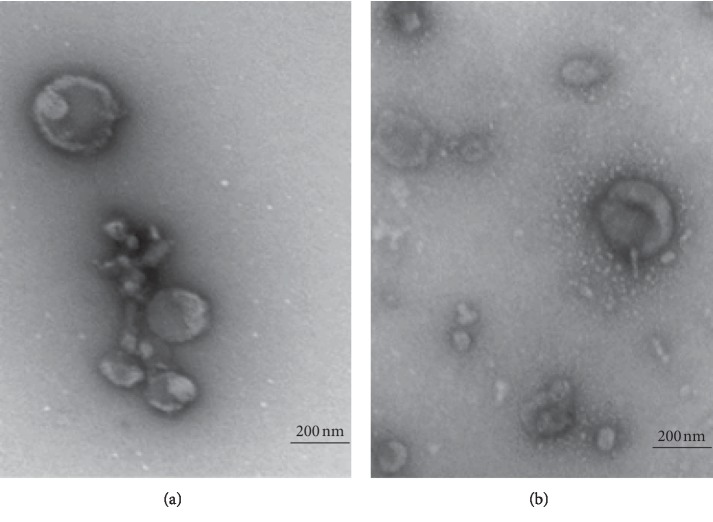
Transmission electron microscopy displaying exosomal morphology and approximate size. Representative electron micrographs of pooled exosome isolations by size exclusion chromatography. (a, b) The presence of intact spherical/donut-shaped particles, for both bovine and human exosomes, respectively. Scale bar 200 nm. (a) Bovine. (b) Human.

A representative TEM image of pooled human exosomes (fractions 7–10) revealed spherical- or donut-shaped morphology and a size range similar to that of bovine exosomes (Figures 4(a) and 4(b)).

### 3.4. Mass Spectrometry

The presence of two tetraspanins (exosomal markers), CD81 and CD9, was observed in both human and bovine milk exosomes.  Table 1 details the peptides that were detected for each tetraspanin in both species, by ProteinPilot™ software.

## 4. Discussion

We previously developed and recently published a method for the isolation and enrichment of exosomes from bovine milk [1]. In the current study, we applied the same methodology for enrichment of exosomes from human milk. This was carried out to access whether the method developed for bovine milk was suitable for use on human milk. Subsequently, the exosomes obtained from human milk were compared to exosomes obtained from bovine milk using three validation methods: exosomal particle concentration by NTA, morphology evaluation by TEM, and the presence of exosomal markers by immunoblotting and IDA mass spectrometry.

Both bovine and human exosomes displayed the presence of FLOT-1 (49 kDa), a well-recognized exosomal marker. Some cross reactivity to the FLOT-1 antibody was seen in the human exosome blots, as bands of approximately 85 kDa, which were absent in bovine exosome blots. CD81 and CD9 were observed in the proteomic results for both human and bovine milk exosomes which is consistent with the literature for both species [5–7]. Several peptides of CD81 and CD9 were found in both species with slight variations in amino acid sequence of the peptides (Table 1). Interestingly, NTA of pooled exosomes identified the mean total yield of exosomes as 1.4 × 10^12^ particles (bovine) and 7.3 × 10^11^ particles (human), in a 2 mL exosome solution. When extrapolated back to the initial volume of milk used, for human milk exosomes, an average particle concentration is comparable to the concentration obtained from bovine milk. No significant differences (using Mann–Whitney *U* test) were obtained between bovine and human samples. However, the particle concentration of human milk exosomes obtained in this study was of two-order magnitude higher than that obtained by Zonneveld et al. [5] and significantly lower than that obtained by Blans et al. [7]. This could be attributed to the differences in the enrichment techniques used [5, 7]. Intact spherical/donut-shaped vesicles were observed for both bovine and human milk exosomes by TEM, consistent with the literature [7].

Although many similarities were observed between exosomes from both species, this study now makes it possible to carry out in-depth comparisons between the two populations. Such comparisons may include miRNA, proteomic and lipidomic profiling, and other functional studies [8–11]. Moreover, the isolation of human milk exosomes with high confidence and purity is of paramount importance for future downstream studies on infant nutrition. Some studies include the comparison of milk obtained from lactating mothers that are on different diets and analysing how the exosomal cargo (proteins and miRNA) varies between the diet groups [8]. This is important for nutritional studies involving breast milk (i.e., nutrition for human babies). Moreover, we can compare the human milk exosomal cargo to that of infant formula to determine the differences in nutritional opportunity. Hence, the study on milk exosomes opens an exciting new and important research platform especially in the field of infant nutrition and metabolism.

In conclusion, the application of the method described in this study for the isolation of exosomes from human milk is comparable to what we have previously described from bovine milk [1]. The enriched exosome profiles between the two species were similar, with the exception of subtle differences in particle number and peptide sequences of the tetraspanins. To date, many methods have been trialed to improve reproducibility and purity of exosomal preparations, using diverse techniques [12–17]. Since a robust method for the isolation of exosomes from milk is required for future downstream applications in both species, our findings suggest a standardized methodology for milk exosome isolation can now be established for human milk exosomes.

## Figures and Tables

**Table 1:** List of peptides observed by IDA mass spectrometry for the tetraspanins CD9 and CD81, from both human and bovine milk exosomes.

	SwissProt ID	ProteinPilot™ nomenclature	Species	Main peptide sequence	Peptide #
Human					
CD9	sp|P21926|CD9_HUMAN	CD9 antigen OS = homo sapiens GN = CD9 PE = 1 SV = 4	Human	AIHYALNCCGLAGGVEQFI KDVLETFTVK	4
CD81	sp|P60033|CD81_HUMAN	CD81 antigen OS = homo sapiens GN = CD81 PE = 1 SV = 1	Human	NNLCPSGSNIISNLFK QFYDQALQQAVVDDDANNAK TFHETLDCCGSSTLTALTT	9

Bovine					
CD9	sp|P30932|CD9_BOVIN	CD9 antigen OS = bos taurus GN = CD9 PE = 2 SV = 2	Bovine	FYEDTYNK NLIDSLK TRPCPEAIDEIFR	3
CD81	sp|Q3ZCD0|CD81_BOVIN	CD81 antigen OS = bos taurus GN = CD81 PE = 2 SV = 1	Bovine	NSLCPSSGNVITNLFK QFYDQALQQAIVDDDANNAK	2

## Data Availability

The data used to support the findings of this study are included within the article and also available upon request. Since large data sets are not available, there is no data repository.
